# Growth Promotion and Disease Suppression Ability of a *Streptomyces* sp. CB-75 from Banana Rhizosphere Soil

**DOI:** 10.3389/fmicb.2017.02704

**Published:** 2018-01-17

**Authors:** Yufeng Chen, Dengbo Zhou, Dengfeng Qi, Zhufen Gao, Jianghui Xie, Yanping Luo

**Affiliations:** ^1^Institute of Tropical Agriculture and Forestry, Hainan University, Haikou, China; ^2^Institute of Tropical Bioscience and Biotechnology, China Academy of Tropical Agricultural Sciences, Haikou, China

**Keywords:** *Streptomyces spectabilis*, banana *Fusarium* wilt, antifungal activity, biosynthetic genes, GC-MS, pot experiments

## Abstract

An actinomycete strain, CB-75, was isolated from the soil of a diseased banana plantation in Hainan, China. Based on phenotypic and molecular characteristics, and 99.93% sequence similarity with *Streptomyces spectabilis* NBRC 13424 (AB184393), the strain was identified as *Streptomyces* sp. This strain exhibited broad-spectrum antifungal activity against 11 plant pathogenic fungi. Type I polyketide synthase (PKS-I) and non-ribosomal peptide synthetase (NRPS) were detected, which were indicative of the antifungal compounds that *Streptomyces* sp. CB-75 could produce. An ethyl acetate extract from the strain exhibited the lowest minimum inhibitory concentration (MIC) against *Colletotrichum musae* (ATCC 96167) (0.78 μg/ml) and yielded the highest antifungal activity against *Colletotrichum gloeosporioides* (ATCC 16330) (50.0 μg/ml). Also, spore germination was significantly inhibited by the crude extract. After treatment with the crude extract of *Streptomyces* sp. CB-75 at the concentration 2 × MIC, the pathogenic fungi showed deformation, shrinkage, collapse, and tortuosity when observed by scanning electron microscopy (SEM). By gas chromatography-mass spectrometry (GC-MS) of the crude extract, 18 chemical constituents were identified; (*Z*)-13-docosenamide was the major constituent. Pot experiments showed that the incidence of banana seedlings was reduced after using *Streptomyces* sp. CB-75 treatment. The disease index was 10.23, and the prevention and control effect was 83.12%. Furthermore, *Streptomyces* sp. CB-75 had a growth-promoting effect on banana plants. The chlorophyll content showed 88.24% improvement, the leaf area, root length, root diameter, plant height, and stem showed 88.24, 90.49, 136.17, 61.78, and 50.98% improvement, respectively, and the shoot fresh weight, root fresh weight, shoot dry weight, and root dry weight showed 82.38, 72.01, 195.33, and 113.33% improvement, respectively, compared with treatment of fermentation broth without *Streptomyces* sp. CB-75. Thus, *Streptomyces* sp. CB-75 is an important microbial resource as a biological control against plant pathogenic fungi and for promoting banana growth.

## Introduction

Phytopathogenic fungi are most worrying, resulting in significant crop yield losses. In addition, some of the fungi produce toxic compounds (Chaiharn et al., 2009). For instance, *Fusarium, Penicillium*, and *Aspergillus* species yield mycotoxins that are harmful to human beings (Almaguer et al., 2012). It is necessary that new and effective methods are sought to prevent phytopathogenic fungi, and to produce crops safe for consumption, as well as to increase crop yield (Law et al., 2017). Due to the increasing incidence of resistance and potential environmental contamination from chemical fungicides, researchers are trying hard to search for novel plant protectants (Wang C. L. et al., 2013). Therefore, it is a good to turn an eye to nature to find antagonistic microorganisms and metabolites (Williams, 2009).

Actinomycetes are one of the most efficient groups of natural bioactive metabolites, and they have been used as antibiotics, antitumor agents, antioxidants, anti-inflammatory agents, anti-infection agents, enzyme inhibitors, pesticides, plant-growth-promoting substances, and so on (Qin et al., 2011; Wang X. J. et al., 2013; Ashokvardhan et al., 2014; Kumar V. et al., 2014; Shivlata and Satyanarayana, 2015; Tan et al., 2016). It has been reported that actinomycetes have been used to protect plants against a wide range of phytopathogenic fungi, and produce cell-wall degrading enzymes, antifungal antibiotics, and plant growth promoters (Yuan and Crawford, 1995; El-Tarabily et al., 2000; Doumbou et al., 2002; Bressan, 2003; Cao et al., 2005; El-Tarabily and Sivasithamparam, 2006; Prapagdee et al., 2008; Jorjandi et al., 2009; Eccleston et al., 2010; Mingma et al., 2014).

Among bioactive compound producers, the genus *Streptomyces* is dominant, and produces compounds such as ivermectin, tetracycline, streptomycin, nystatin, etc. (Ser et al., 2016). *Streptomyces* species are Gram-positive, filamentous, and sporulating actinobacteria, with a high G + C content in their genomes (Lyu et al., 2017). They exhibit an immense biocontrol activity against a range of phytopathogens (Wang X. N. et al., 2013). *Streptomyces* have been long considered simply as free-living soil inhabitants, they can act as plant-growth promoters or as biocontrol agents against soil-borne pathogens (Seipke et al., 2011). *Streptomyces* are regarded as important biological resources, due to their biologically active secondary metabolites; these antimicrobial compounds play roles in protecting plants against pathogens (Ueno et al., 2016). Getha and Vikineswary (2002) found that *Streptomyces violaceusniger* had a strong inhibitory effect on banana *Fusarium* wilt, and a preventative effect (48–52%) on potted plants. *Pseudomonas aeruginosa*, isolated from the banana rhizosphere, has been used as a biological fertilizer to increase banana plant height and reduce vascular discoloration caused by *Fusarium* wilt (Ayyadurai et al., 2006). Therefore, the isolation of antagonistic actinomycetes is considered to be an important step in the development of agriculture, ecosystem safety regulations, and the prevention and control of plant diseases (Lu et al., 2016).

In this study, we isolated and screened *Streptomyces* sp. CB-75 from the soil of a diseased banana plantation. According to 16S rRNA sequence analysis, combined with morphological, culture, physiological, and biochemical characteristics, the taxonomic status of the strain was determined. A preliminary study was performed on the CB-75 strain's antifungal activities against a wide range of fungal pathogens and its effect on potted plants. Also, the antifungal activities of extractions of *Streptomyces* sp. CB-75 were evaluated. Gas chromatography-mass spectrometry (GC-MS) was used to perform chemical analysis of the crude extract of *Streptomyces* sp. CB-75 in order to reveal the chemical constituents present. The aim of this study was to uncover microbial resources, and utilize them in plant protection and microbial fertilizers.

## Materials and methods

### Sampling site and sample collection

Rhizosphere soils, an approximately 10–20 cm layer, were collected from banana plantations in May 2016 in Nanbao (109°51′17″E, 19°47′1″N), Meitai (109°35′58″E, 19°40′51″N), and Huangtong (109°50′58″E, 19°49′58″N) of the Hainan Province, China, transferred into sterile plastic bags using an aseptic metal trowel, and stored at −20°C.

### Test phytopathogenic fungi

The following test phytopathogenic fungi were used in the experiments: *Fusarium oxysporum* f. sp. *cubense* Race 1 (ATCC 76244); *F. oxysporum* f. sp. *cubense* Race 4 (ATCC 76255); *Colletotrichum gloeosporioides* (Penzig) (ATCC 58222); *Colletotrichum fragariae* Brooks (ATCC 58718); *Colletotrichum acutatum* Simmonds (ATCC 56815); *Botrytis cinerea* Persoon (ATCC 11542); *Colletotrichum musae* (ATCC 96167); *Curvulatia fallax* (ATCC 38579); *C. gloeosporioides* (ATCC MYA-456; *Alternaria tenuissima* (ATCC 26513); *C. gloeosporioides* (ATCC 16330). These fungi were provided by the Institute of Environment and Plant Protection, China Academy of Tropical Agricultural Sciences, Haikou, China.

### Isolation of actinomycetes

Actinomycetes were isolated by serial dilution method on Gause's no. 1 medium (Williams et al., 1983; Wang L. Y. et al., 2015). Rifampicin (50 mg/l) and nystatin (50 mg/l) were added to inhibit bacterial and fungal growth. To prepare the soil suspension, 10 g of the dried soil sample was transferred into a 250-ml bottle, and 90 ml sterile distilled water was added to the bottle, with shaking for 30 min. Soil suspensions from 10^−3^ to 10^−5^-fold dilutions were aseptically plated onto Gause's no. 1 synthetic medium and incubated at 28°C for 7–15 days. Colonies with different morphological characteristics was transferred and purified on yeast extract-malt extract (ISP 2) agar (Shirling and Gottlieb, 1966) at 4°C, and kept for long term preservation in 20% (v/v) glycerol at −80°C (Williams et al., 1983).

### Screening of actinomycetes

Antagonistic activities of purified strains were determined by the conventional spot inoculation method (Sadeghian et al., 2016; Sharma et al., 2016), and their extracts with ethyl acetate (EtOAc) were tested using the agar diffusion method, according to Patel et al. (2011) with slight modifications. Actinomycete cakes (Φ = 5 mm) were inoculated onto one side of potato dextrose agar (PDA) plates (Wang L. Y. et al., 2015), about 2.5 cm from the center of the plate. A phytopathogenic fungal disc (Φ = 5 mm) was placed in the center of the plate. A fungal disc alone in the center of a plate served as a control. After incubation at 28°C for 5–7 days, the antagonistic belt (inhibition zone) was recorded by measuring the distance between the edge of the fungal mycelium and the actinomycete discs. The percentage inhibition of the radial growth (PIRG) was calculated using the following formula:

$$\begin{array}{l} \text{PIRG }=\;\left[\left(R1-R2\right)/R1\right]\times 100 \end{array}$$

where *R*1 was the radius of fungal mycelial growth in the control, and *R*2 was the radius of fungal mycelial growth that occurred toward the actinomycetes. All strains were tested in three independent experiments (Albuquerque et al., 2006).

### Identification and characterization of the actinomycete strain

#### Culture characteristics

The culture characteristics of strain CB-75 were examined according to the method of Shirling and Gottlieb (1966). The strain was grown in different culture media (ISP2, ISP3, ISP4, ISP5, ISP6, ISP7, PDA, and Gause's no. 1) (Shirling and Gottlieb, 1966) at 28°C for 7–10 days. Colony colors were distinguished with the ISCC-NBS color charts (Kelly, 1958), and the colony characteristics, including growth, aerial mycelium, spores, and hypha characteristics, were observed. Morphological characteristics were observed under scanning electron microscopy (SEM) (SIGMA Field Emission Scanning Electron Microscope). Growth at various temperatures (4–45°C) was determined on ISP2 at 28°C for 14–21 days. The pH (3–11) and NaCl (0–15%) tolerance for growth were determined on ISP2 at 28°C for 14–21 days. The utilization of carbohydrates as sole carbon sources was tested according to published methods (Gordon et al., 1974). Decomposition of casein, tyrosinase production, H2S production, gelatin liquefaction, nitrate reduction, and starch hydrolysis were assessed according to the reference by Williams et al. (1983). Major diagnostic cell wall sugars of *Streptomyces* sp. CB-75 were obtained as described by Whiton et al. (1985).

### Molecular identification

#### DNA extraction and PCR amplification

The preparation of genomic DNA of the strain was implemented in accordance with the methods described by Pitcher et al. (1989). The 16S rRNA gene was amplified by PCR with *Taq* DNA polymerase and the conserved primers 27F (5′-AGAGTTTGATCMTGGCTCAG-3′) and 1492R (5′-TACGGYTACCTTGTTACGACT-3′) (Gupta et al., 2014). PCRs were performed in the TProfessional Trio PCR System (Biometra, Germany). The PCR system and conditions were as described by Himaman et al. (2016). The PCR amplification products were visualized by 1.0% (w/v) agarose gel electrophoresis. The amplified PCR products were sequenced by a Sanger-based, automated sequencer (Applied Biosystems).

#### Phylogenetic analysis

The almost-complete 16S rRNA gene sequence was compared with those deposited in public databases and the EzBiocloud server (https://www.ezbiocloud.net/identify; Chun et al., 2007; Kim et al., 2012), which was also used to calculate pairwise sequence similarities. The 16S rRNA gene sequence of representative related taxa were obtained from the GenBank databases using CLUSTAL_X software (Thompson et al., 1997). The alignment was manually verified and adjusted prior to the reconstruction of a phylogenetic tree. The phylogenetic tree was reconstructed with the neighbor-joining tree algorithm (Saitou and Nei, 1987) and maximum parsimony method (Fitch, 1971) using MEGA version 7.0 (Tamura et al., 2007). The evolutionary distances of the clades in the neighbor-joining tree were estimated by bootstrap analysis (Felsenstein, 1985).

### Antifungal activity assay

#### EtOAc extraction

*Streptomyces* sp. CB-75 was inoculated in 1,000 ml of sterilized soybean liquid culture medium (SLM) (Hong et al., 2009), and incubated with shaking (150 rpm) at 28°C for 7 days. Then, the culture filtrate was extracted with EtOAc at a ratio of 1:1 (v/v). The mixture was filtered through Whatman no. 1 qualitative filter paper and the organic phase (EtOAc) was separated from the filtered liquid media using a decantation funnel. The extraction was evaporated in a rotary vacuum evaporator. The crude extract of *Streptomyces* sp. CB-75 was prepared by dissolving in 10% dimethyl sulfoxide (DMSO) at a concentration of 20.0 mg/ml, and sterilized by filtration through a 0.22 μm sterile filter (Millipore, Bedford, MA, USA). The extract were stored at −4°C.

#### Antifungal activity by disc diffusion assay

To assess the antifungal activity of the crude extract of *Streptomyces* sp. CB-75 against 11 indicator plant pathogens, bioassays were carried out using the disc diffusion method (Ashokvardhan and Satyaprasad, 2016; Sharma et al., 2016). Sterilized filter paper discs (Φ = 6 mm) were impregnated with the crude extract and placed on PDA plates about 3 cm from the center of the plate. A fungal disc (Φ = 5 mm) was placed in the center of the plate. A fungal mycelia disc alone in the center of the plate served as a control. The diameters of the inhibition zones were measured after incubation for 7 days at 28°C. The inhibition zone was equal to R1–R2 (Himaman et al., 2016), where R1 was the radius of fungal mycelial growth in the control and R2 was the radius of fungal mycelial growth that occurred toward *Streptomyces* sp. CB-75. Three replicates were performed for each treatment, and each assay was repeated three times.

#### Antifungal activity on mycelia radial growth

The antifungal activity of the crude extract of *Streptomyces* sp. CB-75 on mycelial growth was assessed by the poisoned food technique (Sharma et al., 2016). The crude extract, dissolved in 10% DMSO (20.0 mg/ml), was added to PDA medium at 45–50°C to get 100 μg/ml. PDA containing the crude extract (20 ml) was poured into sterilized Petri dishes (90 mm in diameter). An equal amount of 10% DMSO was used as a control. A fungal disc (5 mm in diameter) was inoculated aseptically into the center of each Petri dish. The plates were sealed with polyethylene film, and incubated at a temperature of 28 ± 2°C until the control mycelium reached the edge of the plates. The mean of the perpendicular diameters of each colony was measured. Each assay was repeated three times. The percentage inhibition of the mycelial radial growth was calculated using the following formula:

$$\begin{array}{l} \text{Percentage Mycelial Inhibition }=\;\frac{\text{C }-\text{ T}}{\text{C}}\times 100 \end{array}$$

where C is the mean colony diameter for the control, and T is the mean colony diameter for the treatment (Nimaichand et al., 2015).

#### PCR amplification and sequencing of biosynthetic genes of *Streptomyces* sp. CB-75 [genes encoding type I polyketide synthases (PKS-I) and non-ribosomal peptide synthetases (NRPSs)]

Two sets of degenerate PCR primers (K1F 5′-TSAAGTCSAACATCGGBCA-3′ and M6R 5′-CGCAGG TTSCSGTACCAGTA-3′) targeting PKS-I sequences were used for amplification of ketosynthase and methyl-malonyl transferase domain sequences (Gonzalez et al., 2005). A3F (5′-GCSTACSYSATSTACACSTCSGG-3′) and A7R (5′-SASGTCVCCSGTSCGGTAS-3′) were used for amplification of NRPS adenylation domain sequences (Sharma et al., 2016). PKS-I and NRPS PCR amplifications were performed with a TProfessional Trio PCR System (Biometra, Germany), in a total volume of 25 μl consisting of template DNA (50 ng), 2 × PCR Master Mix (12.5 μl), DDH_2_O (10.5 μl), K1F/A3F (0.5 μl), and M6R/A7R (0.5 μl). The conditions for thermal cycling were as follows: denaturation of the DNA at 95°C for 5 min; and 35 cycles at 94°C for 30 s, primer annealing 2 min at 55°C for K1F/M6R and 59°C for A3F/A7R, and DNA elongation at 72°C for 4 min. At the end of the PCR, the reaction mixture was held at 72°C for 10 min (Himaman et al., 2016). PCR amplification products were analyzed by electrophoresis in 1% (w/v) agarose gel stained with goldview I. The resultant sequences were compared with other known sequences in GenBank by available BLAST methods (http://www.ncbi.nlm.nih.gov/BLAST/).

#### Determination of the minimum inhibitory concentration (MIC) of *Streptomyces* sp. CB-75

A 96-well microtiter assay (Wedge et al., 2003; Wang X. N. et al., 2013) was used to determine the MICs of the crude extract from *Streptomyces* sp. CB-75 against the test fungal pathogens in comparison with known fungicides. Different concentrations of the crude extract were prepared using two-fold serial dilutions for MIC tests, the final concentrations were 50.0, 25.0, 12.5, 6.25, 3.125, 1.563, and 0.781 μg/ml. The lowest concentration of the crude extract that inhibited growth was recorded as the MIC. Each well received 80 μl of Roswell Park Memorial Institute (RPMI) mycological media, 100 μl of test fungi conidia at 1.0 × 10^5^ CFU/ml, and 20 μl of antifungal solution from the parent plate. Each test extract was evaluated in duplicate against a non-inoculated well (reagent blank) containing test extract and RPMI at each concentration. The 96-well plates (Nunc MicroWell, untreated; Roskilde, Denmark) were covered with a plastic lid and incubated at 24 ± 1°C for a 12 h photoperiod under 60 ± 5 μmol light. The absorbance was measured at 620 nm using a microplate photometer (Packard Spectra Count, Packard Instrument Co., Downers Grove, IL, USA). A negative control was prepared using 10% DMSO, and standard antibiotics such as cycloheximide and nystatin were used as positive controls.

#### Spore germination assay

The percentage spore germination was calculated using the method of Tzortzakis and Economakis (2007), with slight modifications. Each fungal strain was challenged in a dose-response format using the crude extract of *Streptomyces* sp. CB-75, where the final treatment concentrations were 1 × MIC, 2 × MIC, 4 × MIC, and 8 × MIC. Each extract solution was mixed with the fungal spore suspension (10^5^ CFU) at a ratio of 1:1 (v/v). The mixture (0.1 ml) was placed on a sterile glass slide. Slides containing the spores were incubated in a moist chamber at 28°C for 20 h. A mixture of sterile water and spores was used as a control. Each treatment was repeated three times. Spore germination was observed by electron microscopy (mag = 200 × lens). For each treatment, one hundred spores were examined, and the extent of spore germination assessed by looking for germ tube emergence. The number of germinated spores was scored using a hemocytometer, and the percentage of spore germination (PSG) was calculated as follows:

$$\begin{array}{l} PSG=\frac{\text{A }-\text{ B}}{\text{A}}\times 100, \end{array}$$

where A was the spore germination rate of the control group, and B was the spore germination rate of treatment group (Sharma et al., 2017).

#### SEM

After treatment with the crude extract of *Streptomyces* sp. CB-75, the cells of *F. oxysporum* f. sp. *cubense* Race 4 were observed by SEM according to a previously published method (Supaphon et al., 2013), with slight modifications. The inoculum was prepared by growing the test pathogen for 5~7 days. Conidial suspensions were prepared according to published procedures (Wedge and Kuhajek, 1998). Conidial concentrations were determined photometrically (Espinel-Ingroff and Kerkering, 1991; Wedge and Kuhajek, 1998) from a standard curve and suspensions were then adjusted with sterile distilled water to a concentration of 1.0 × 105 conidia/ml. Conidia were then treated with the crude extract of CB-75 at the 2 × MIC value for 24 h. The cells were fixed with 2.5% (v/v) glutaraldehyde (C3H8O2) in phosphate-buffered saline (PBS) for 2 h, and washed with PBS (pH 7.4) and water. The cells were dehydrated with series of increasing concentrations of alcohol (30, 50, 70, 80, 90, and 100%) for 20 min. Finally, the ethanol was displaced with isoamyl acetate. The cells were dried for 30 min and mounted onto a steel stub with double-sided carbon tape. Samples were coated with a film of gold-palladium alloy under vacuum and observed with a scanning electron microscope (Zeiss Sigma VP, Germany).

#### GC–MS analysis

GC-MS analysis was performed to identify the chemical compounds in the crude extract of *Streptomyces* sp. CB-75, in accordance with a previously described method, with slight modifications (Sun et al., 2015; Supriady et al., 2015). GC-MS analysis was performed on a Shimadzu GC 2010 plus with triple quadrupole mass spectrometer (TP-8030), and fitted with a DB-5ms (5% phenyl methyl siloxane) capillary column of dimensions 30 m × 0.25 mm × 0.25 μm, and with helium as a carrier gas at 1 ml/min.

The column temperature was programmed initially at 60°C, held for 1.0 min, and then increased to 100°C at 5.0°C/min, held for 5.0 min, and then again raised at 10.0°C/min to 250°C, held for 35.0 min, and finally raised to 280°C at 10.0°C/min, and held for 25.0 min. The mass spectrometer was operated in electron ionization (EI) mode at 70 eV, with an interface temperature of 280°C, an ion source temperature of 240°C, a mass spectrometer acquisition delay time of 3.5 min, and a continuous scan from 50 to 650 amu. Peaks were identified by comparison with the mass spectra data from the National Institute of Standards and Technology (NIST) spectral library.

#### Pot culture experiments

Pot culture experiments were carried out in May-July 2017 at the Institute of Tropical Bioscience and Biotechnology, China Academy of Tropical Agricultural Sciences. The conditions of the greenhouse were 28°C, 70% humidity, and with natural light. The fermentation broth of *Streptomyces* sp. was inoculated in sterilized soybean liquid culture medium (SLM), and incubated with shaking (150 rpm) at 28°C for 7 days. The fermentation broth was filtered through two layers of sterile prewetted Mira cloth. The filtrate was diluted 50-fold, and 100 ml liquid put into each plastic pot. Five treatment groups were established: CK1 (non-inoculated *F. oxysporum* Race 4 and application of sterile water); CK2 (inoculated *F. oxysporum* Race 4 and application of sterile water); CK3 (inoculated *F. oxysporum* Race 4 and sodium *p*-(dimethylamino) benzenediazo sulfonate); A (inoculated *F. oxysporum* Race 4 and application of fermentation broth without *Streptomyces* sp. CB-75); AB (inoculated *F. oxysporum* Race 4 and fermentation broth of *Streptomyces* sp. CB-75).

Soil was collected from Lingao county, passed through a twenty-mesh sieve, and sterilized at 160°C for 2 h, and then 700 g was packaged into each plastic pot. Banana seedlings were washed with sterile water, and the second piece of the taproot cut. The plants were aseptically planted into the plastic pots with 700 g potting soil. The fungal spore suspension of *F. oxysporum* Race 4 was inoculated into the potting soil, and diluted at the concentration of 10^5^ CFU/g soil. The banana seedlings were cultivated in a greenhouse. Each experiment was repeated three times.

The grading standards of *F. oxysporum* f. sp. *cubense* Race 4, the disease index, and the effect of prevention and control were shown as follows (Himaman et al., 2016):

grade 0, healthy plant;
grade 1, the leaves of the lower part of the plant were withered;
grade 3, 20–40% of the leaves were withered;
grade 4, 40–60% of the leaves were withered;
grade 5, 60–80% of the leaves were withered;
grade 6, the entire plant was withered and dead.

$$\begin{array}{ll} \text{Disease Index} & =\frac{\sum \left(\text{Number of diseased plants of each grade }\times \text{ value of relative rade}\right)}{\text{Total number inspected }\times \;6}\times 100\\ \text{Controlling effect }\left(\text{\%}\right) & =\;\frac{\text{Sterile water controlled disease index}-\text{treated disease index}}{\text{Sterile water controlled disease index}}\times 100 \end{array}$$

#### Physiological indexes of banana

The physiological indexes of banana seedlings transplanted at 0, 15, 30, 45, and 60 days were determined, including chlorophyll content, leaf area, root length, root diameter, plant height, stem, shoot biomass, and root biomass on the 60th day.

#### Statistical analysis

Analysis of variance and multiple comparisons were performed using SAS 6.12 software. All experiments were performed in biological triplicate and repeated three times. The data were expressed as the mean ± standard deviation of the mean of the three replicates by variance analysis of single factor analysis. Duncan's multiple range test was performed at a significance level of *P* < 0.05.

## Results

### Isolation of actinomycetes from the rhizosphere soil

One hundred and thirty morphologically different actinomycetes strains were isolated from the soil of a diseased banana plantation in Hainan, China. Soil niches have been reported to be rich in many significant actinomycetes (Savic et al., 2007; Bizuye et al., 2013; Tan et al., 2015). All isolates were screened for their antifungal activity using a conventional spot inoculation method and the agar diffusion method. Out of all of them, 14 per cent of the isolates exhibited antifungal activity during the preliminary experiment, especially strain CB-75, which exhibited broad-spectrum antifungal activity against the tested phytopathogenic fungi. So, *Streptomyces* sp. CB-75 was selected and identified.

### Characterization of *Streptomyces* sp. CB-75

#### Culture characteristics

**Figure 1:**
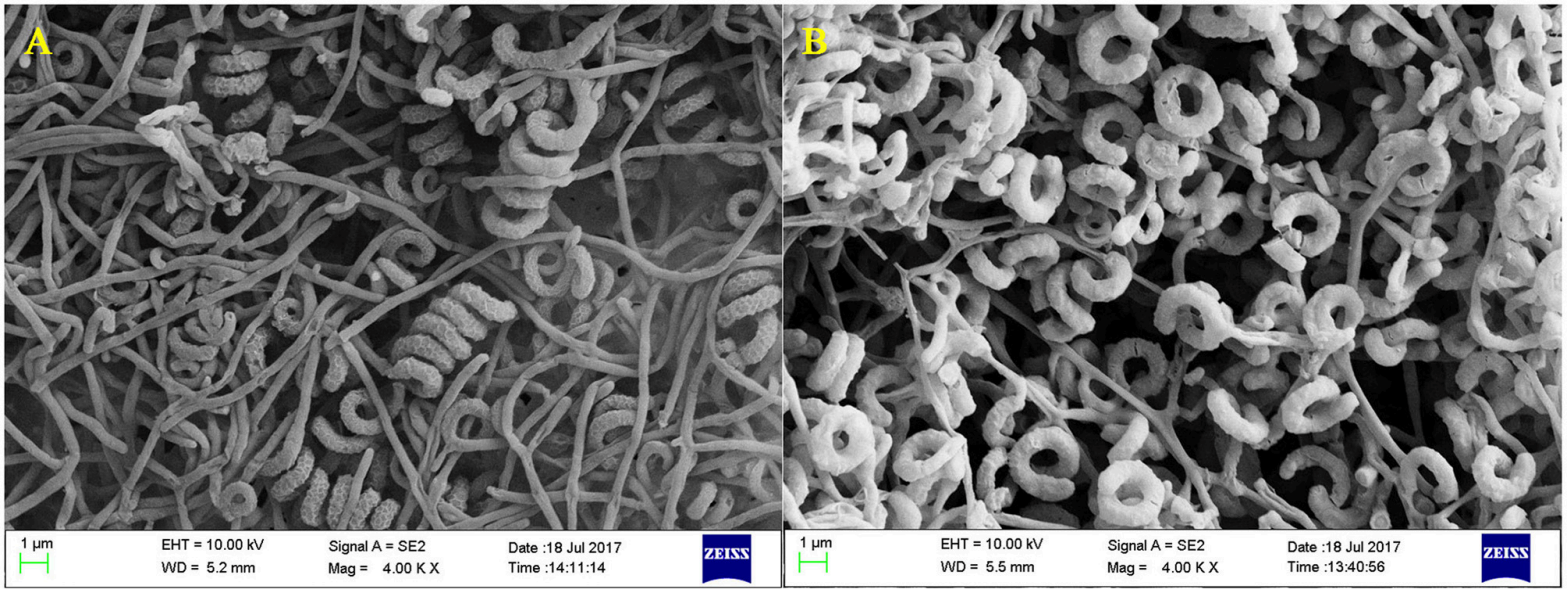
Scanning electron microscope images of *Streptomyces* sp. CB-75. Morphological characters of aerial mycelia **(A)** and spores **(B)** of *Streptomyces* sp. CB-75 viewed using SEM.

*Streptomyces* sp. CB-75 was cultured on different media, and its aerial mycelium, substrate mycelium, and soluble pigments were assessed (Table 1). *Streptomyces* sp. CB-75 grew very well on 8 types of culture media, and did not produce soluble pigments. The aerial mycelia were straight and long under SEM analysis (Figure 1A). It formed spiral chains of rugose ornamented spores (Figure 1B).

**Table 1:** Culture characteristics of *Streptomyces* sp. CB-75.

**Medium**	**Aerial mycelium**	**Vegetative mycelium**	**Soluble pigment**	**Growth Growth**
ISP 2	Light brown	Red-orange	None	+++
ISP 3	Yellow-pink	Red-orange	None	++
ISP 4	White	Dark pink	None	+++
ISP 5	Orange-pink	Orange-pink	None	+
ISP 6	Pink	Dark pink	None	+++
ISP 7	Orange	Orange	None	++
PDA	Pink	Pink	None	++
Gause's no. 1 agar	Orange-yellow	Red	None	+++

*+++, Good growth; ++, Moderate growth; +, Poor growth*.

#### Physiological and biochemical characteristics

The physiological and biochemical characteristics of *Streptomyces* sp. CB-75 have been tabulated into Table 2. *Streptomyces* sp. CB-75 could reduce nitrate, decompose sulfur-containing amino acids to H_2_S, and was able to hydrolyze starch and produce melanin. However, *Streptomyces* sp. CB-75 was unable to liquefy gelatin, nor peptonize and solidify milk, and could not produce tyrosinase. It could fully utilize α-lactose, D-cellobiose, D-fructose, D-galactose, D-glucose, D-mannose, D-sorbitol, D-trehalose, LL-arabinose, melitose, melibiose, xylan, D-mannitol, melezitose, ribose, saligenin, and soluble starch as carbon sources. Furthermore, the stain could fully utilize L-arginine, L-phenylalanine, glycine, methionine, L-hydroxyproline, L(+)-cysteine, L-homocysteine, valine, histidine, ammonium nitrate, and ammonium chloride as nitrogen sources. It grew at 18~45°C, pH 6~9, and its optimal growth temperature was 28°C and pH 7.2. It grew well in media with less than 5% NaCl. Its cell wall contained both LL-diaminopimelic acids (DAP) and glycine based on cellulose thin layer chromatography (TLC) analysis, the cell wall of *Streptomyces* sp. CB-75 belonged to type I, and its whole cell sugar belonged to type C.

**Table 2:** Physiological and biochemical characteristics of *Streptomyces* sp. CB-75.

**Characteristic**	**Result**
**BIOCHEMICAL TEST**	
Gelatin liquefaction	−
Milk peptonization	−
Milk solidification	−
Nitrate reduction	+
Amylolysis	+
H2S	+
Pigment	+
Tyrosinase	−
**NITROGEN SOURCE UTILIZATION**	
L-Arginine	+
L-Serine	−
L-Phenylalanine	+
Glycine	+
Methionine	+
L-Hydroxyproline	+
L(+)-Cysteine	+
L-Homocysteine	+
Valine	+
Histidine	+
Ammonium nitrate	+
Ammonium chloride	+
**CARBON-SOURCE UTILIZATION**	
α-Lactose	+
D-Cellobiose	+
D-Fructose	+
D-Galactose	+
D-Glucose	+
D-Mannose	+
D-Sorbitol	+
D-Trehalose	−
D-Xylose	+
L-Arabinose	+
Melitose	+
Melibiose	+
Xylan	+
D-Mannitol	+
Inositol	−
Melezitose	+
Rhamnose	−
Ribose	+
Saligenin	+
Soluble starch	+
Sucrose	−

*+, Positive reaction; −, Negative reaction*.

#### Identification and phylogenetic analysis of isolates

**Figure 2:**
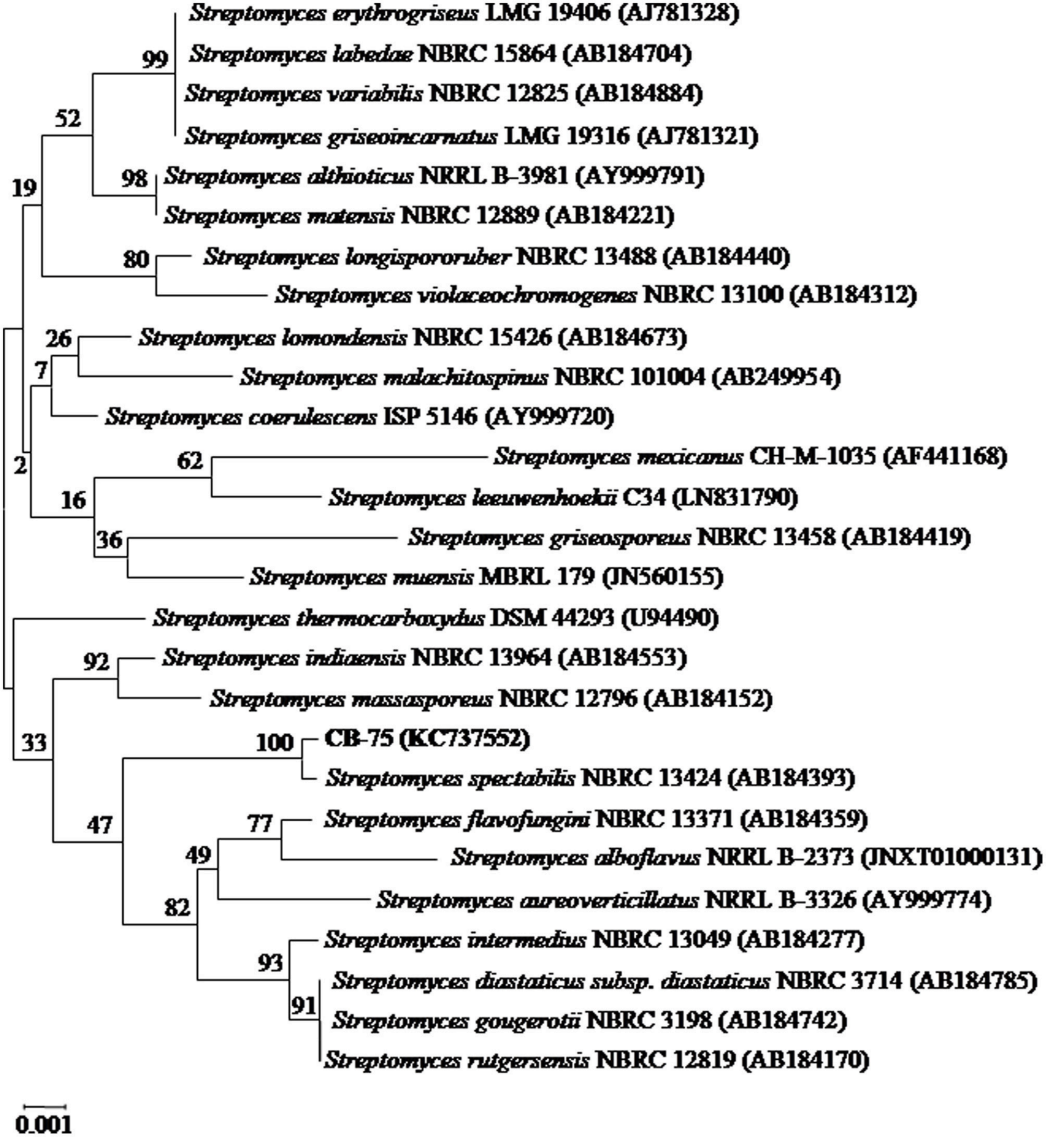
Neighbor-joining phylogenetic tree based on almost complete 16S rRNA gene sequences (1,490 nucleotides) showing the phylogenetic relationship between strain *Streptomyces* sp. CB-75 and representatives of some other related taxa. Bootstrap percentages based on 1,000 resamplings are listed at nodes, only values above 50% are shown. GenBank accession numbers are given in parentheses. Bar, 0.001 substitutions per nucleotide position.

A partial 16S rRNA gene sequence (1,490 nucleotides) of strain CB-75 was determined and submitted to the GenBank database under the accession number KC737552. The strain exhibited the highest similarity with *Streptomyces spectabilis* NBRC 13424 (AB184393; 99.93%), using EzBiocloud and GenBank sequence similarity searches and homology analysis. A phylogenetic tree was reconstructed with the neighbor-joining and maximum-parsimony methods, using the software package MEGA version 7.0 (Figure 2). Phylogenetic analysis demonstrated that strain CB-75 was closely related to *Streptomyces spectabilis* NBRC 13424 (AB184393; 99.93%), as they formed a distinct clade at a high bootstrap value of 100% (Figure 2). The phenotypic (morphological, physiological, and biochemical characteristics) and genomic data were indicative that strain CB-75 was representative of members of the genus *Streptomyces*. The strain was referred to as *Streptomyces* sp. CB-75.

#### Antifungal activity assay of *Streptomyces* sp. CB-75

The crude extract from *Streptomyces* sp. CB-75 showed a wide antifungal spectrum (Table 3). It exhibited excellent inhibitory activity against 11 pathogenic fungi. The best inhibitory activity (the inhibition zones ± SD mm) was found against *C. musae* (ATCC 96167) (inhibition zone diameter of 14.93 ± 0.35) and *C. acutatum* (ATCC 56815) (14.43 ± 0.40), followed by *F. oxysporum* Race 4 (ATCC 76255) (13.77 ± 0.31), *C. fallax* (ATCC 38579) (13.67 ± 0.31), *C. gloeosporioides* (ATCC 58222) (13.50 ± 0.50), *B. cinerea* (ATCC 11542) (13.23 ± 0.25), and *C. gloeosporioides* (ATCC MYA-456) (13.23 ± 0.38). There was no significant difference with the inhibitory zones (*P* < 0.05). The minimal inhibition zone was 11.40 ± 0.36 mm against *F. oxysporum* Race 1 (ATCC 76244).

**Table 3:** Inhibitory action of *Streptomyces* sp. CB-75 against plant pathogenic fungal strains.

**Pathogenic fungal**	**Inhibition zone (mm)**	**Mycelial inhibition (%)**
*F. oxysporum* Race 1 (ATCC 76244)	11.40 ± 0.36 d	73.11 ± 0.80 d
*F. oxysporum* Race 4 (ATCC 76255)	13.77 ± 0.31 b	78.37 ± 0.68 b
*C. gloeosporioides* (ATCC 58222)	13.50 ± 0.50 b	77.78 ± 1.11 b
*C. fragariae* (ATCC 58718)	12.83 ± 0.29 c	76.30 ± 0.64 c
*C. acutatum* (ATCC 56815)	14.43 ± 0.40 a	79.85 ± 0.90 a
*B. cinerea* (ATCC 11542)	13.23 ± 0.25 bc	77.19 ± 0.56 bc
*C. musae* (ATCC 96167)	14.93 ± 0.35 a	80.96 ± 0.78 a
*C. fallax* (ATCC 38579)	13.67 ± 0.31 b	78.15 ± 0.68 b
*C. gloeosporioides* (ATCC MYA-456)	13.23 ± 0.38 bc	77.19 ± 0.84 bc
*A. tenuissima* (ATCC 26513)	11.73 ± 0.38 d	73.85 ± 0.84 d
*C. gloeosporioides* (ATCC 16330)	12.70 ± 0.36 c	76.00 ± 0.80 c

*Data in the table are means ± SD. Different lowercase letters in the same column show values that are significantly different at the P < 0.05 level by Duncan's new multiple range test*.

*Streptomyces* sp. CB-75 inhibited mycelial growth of 11 types of pathogenic fungi. The percentage inhibition of the mycelial radial growth values varied greatly among the target species, ranging from 73.11 to 80.96%. The maximum percentage of mycelial growth inhibition was against *C. musae* (ATCC 96167) (80.96 ± 0.78%) and *C. acutatum* (ATCC 56815) (79.85 ± 0.90%). The minimum was against *A. tenuissima* (ATCC 26513) (73.85 ± 0.84%) and *F. oxysporum* Race 1 (ATCC 76244) (73.11 ± 0.80%). Thus, *Streptomyces* sp. CB-75 showed a wide range of antifungal activity.

#### Identification of biosynthetic genes (encoding PKS-I and NRPS)

It is well known that PKSs and NRPSs are biosynthetic enzymes, which induce the formation of active metabolites in actinomycetes (Hodges et al., 2012). It has been helpful to evaluate the biosynthetic potential of actinobacteria through identification of biosynthetic genes (Nimaichand et al., 2015). For PCR amplification of PKS-I-encoding genes, K1F/M6R primers were used corresponding to PKS-I ketosynthase and methyl-malonyl-CoA transferase modules, the strain CB-75 had a band size of approximately 1,200–1,400 bp. Sequencing of the gene fragment encoding PKS-I yielded a sequence of 1234 bp (National Center for Biotechnology Information [NCBI] accession no. MF476983). Strain CB-75 showed the highest sequence similarity (84%) for the PKS-I-encoding gene with a PKS-encoding gene from *Streptomyces alboflavus* strain MDJK44 (NCBI accession no. CP021748). The NRPS amplicon was found to be 600–700 bp in size, following amplification with A3F/A7R specific primers for NRPS adenylation domain sequences. Sequencing of the NRPS-encoding gene fragment yielded a sequence of 651 bp in length (NCBI accession no. MF476984). The NRPS sequence of CB-75 showed the highest similarity (96%) to an NRPS-encoding gene from *Streptomyces albus* strain NK660 (NCBI accession no. CP007574).

#### MIC of *Streptomyces* sp. CB-75

The MIC values of the crude extract from *Streptomyces* sp. CB-75 were further determined against all tested pathogenic fungi using a 96-well microtiter assay. The MIC values of the crude extract were found within the range of 50–0.781 μg/ml. The lowest inhibitory concentration of the crude extract against these fungi was assessed as the MIC. The lowest MIC was 0.781 μg/ml against *C. musae* (ATCC 96167), which showed the crude extract yielded a strong antifungal activity against this strain. However, the highest MIC was 50.0 μg/ml against *C. gloeosporioides* (ATCC 16330) (Table 4). The 10% DMSO control had no inhibitory effect on tested pathogenic fungi.

**Table 4:** MIC values (μg/ml) from the microtiter assay for *Streptomyces* sp. CB-75 by broth dilution method.

**Pathogenic fungi**	**MIC of CB-75 (μg/ml)**	**MIC of Cy (μg/ml)**	**MIC of Az (μg/ml)**
*F. oxysporum* Race 1 (ATCC 76244)	>6.25	>6.25	>12.5
*F. oxysporum* Race 4 (ATCC 76255)	>3.125	>6.25	>25.0
*C. gloeosporioides* (ATCC 58222)	>1.563	>3.125	>3.125
*C. fragariae* (ATCC 58718)	>6.25	>6.25	>6.25
*C. acutatum* (ATCC 56815)	>3.125	>0.781	>1.563
*B. cinerea* (ATCC 11542)	>1.563	>12.5	>6.25
*C. musae* (ATCC 96167)	>0.781	>1.563	>3.125
*C. fallax* (ATCC 38579)	>3.125	>3.125	>6.25
*C. gloeosporioides* (ATCC MYA-456)	>12.5	>12.5	>25.0
*A. tenuissima* (ATCC 26513)	>12.5	>12.5	>12.5
*C. gloeosporioides* (ATCC16330)	>50.0	>25.0	>50.0

*Cy, Cycloheximide (antifungal agent); Az, Azoxystrobin (antifungal agent)*.

#### Spore germination assay

**Figure 3:**
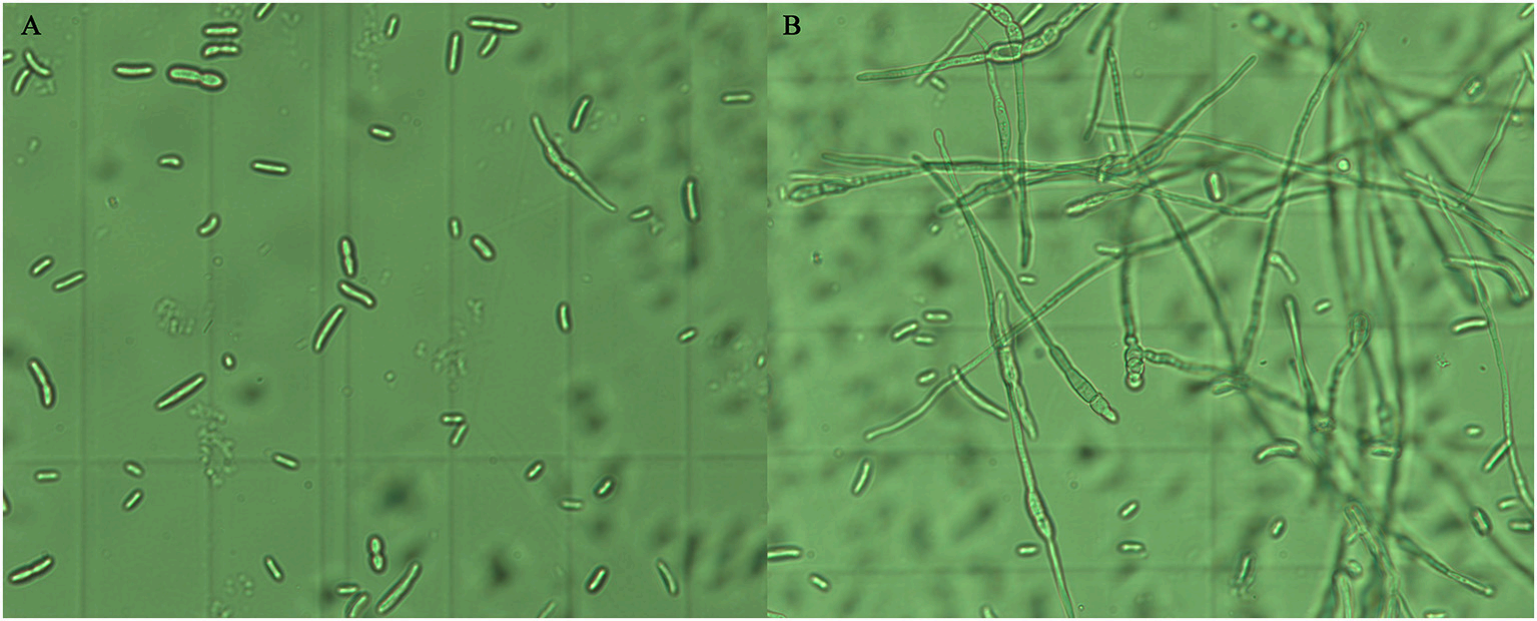
Inhibition effect of the crude extract of *Streptomyces* sp. CB-75 on spore germination of *F. oxysporum* Race 4 (ATCC 76255). **(A)** Treatment with the crude extract of *Streptomyces* sp. CB-75; **(B)** without treatment.

The effects of the crude extract of *Streptomyces* sp. CB-75 on spore germination of the tested pathogenic fungi are shown in Figure 3. The results show that spore germination was significantly different (*P* < 0.05) with different concentrations of the crude extract. The percentage of spore germination decreased with increasing concentration of the crude extract. The concentration of 8 × MIC was most effective against spore germination (Table 5). The maximum percentage of spore germination inhibition was 93.96% against *C. musae* (ATCC 96167). The minimum percentage of spore germination inhibition was 83.12% against *A. tenuissima* (ATCC 26513). The 10% DMSO (v/v), as a control, did not inhibit the spore germination of the tested pathogenic fungi.

**Table 5:** The impact of the crude extract of *Streptomyces* sp. CB-75 on spore germination of the test pathogenic fungi.

**Pathogenic fungi**	**Inhibition of spore germination (%)**			
	**1 × MIC**	**2 × MIC**	**4 × MIC**	**8 × MIC**
*F. oxysporum* Race 1 (ATCC 76244)	44.97 ± 0.88 g	66.37 ± 1.24 h	75.62 ± 0.91 g	86.13 ± 1.11 g
*F. oxysporum* Race 4 (ATCC 76255)	55.23 ± 1.76 b	69.95 ± 1.01 c	79.57 ± 1.07 b	91.37 ± 0.81 c
*C. gloeosporioides* (ATCC 58222)	55.24 ± 1.62 b	69.67 ± 0.84 c	79.45 ± 0.92 b	91.22 ± 1.34 c
*C. fragariae* (ATCC 58718)	53.91 ± 1.35 c	67.35 ± 0.72 f	76.74 ± 0.72 e	90.20 ± 1.03 d
*C. acutatum* (ATCC 56815)	54.20 ± 1.45 c	68.61 ± 1.15 d	78.80 ± 0.95 c	93.43 ± 0.95 b
*B. cinerea* (ATCC 11542)	52.95 ± 1.39 d	66.82 ± 0.86 g	77.10 ± 0.73 d	89.63 ± 1.18 e
*C. musae* (ATCC 96167)	57.48 ± 1.50 a	73.30 ± 1.15 a	81.25 ± 0.97 a	93.96 ± 0.99 a
*C. fallax* (ATCC 38579)	52.85 ± 0.98 d	71.09 ± 0.74 b	78.58 ± 1.40 c	87.14 ± 0.93 f
*C. gloeosporioides* (ATCC MYA-456)	46.27 ± 0.98 f	67.87 ± 1.03 e	70.95 ± 1.03 i	84.25 ± 0.80 h
*A. tenuissima* (ATCC 26513)	39.26 ± 0.79 h	60.27 ± 1.40 i	73.01 ± 0.85 h	83.12 ± 1.16 i
*C. gloeosporioides* (ATCC 16330)	48.28 ± 1.25 e	67.53 ± 1.08 ef	76.26 ± 1.11 f	87.22 ± 0.75 f

*Data in the table are means ± SD. Different lowercase letters in the same column show values that are significantly different at the P < 0.05 level by Duncan's new multiple range test*.

#### SEM analysis

**Figure 4:**
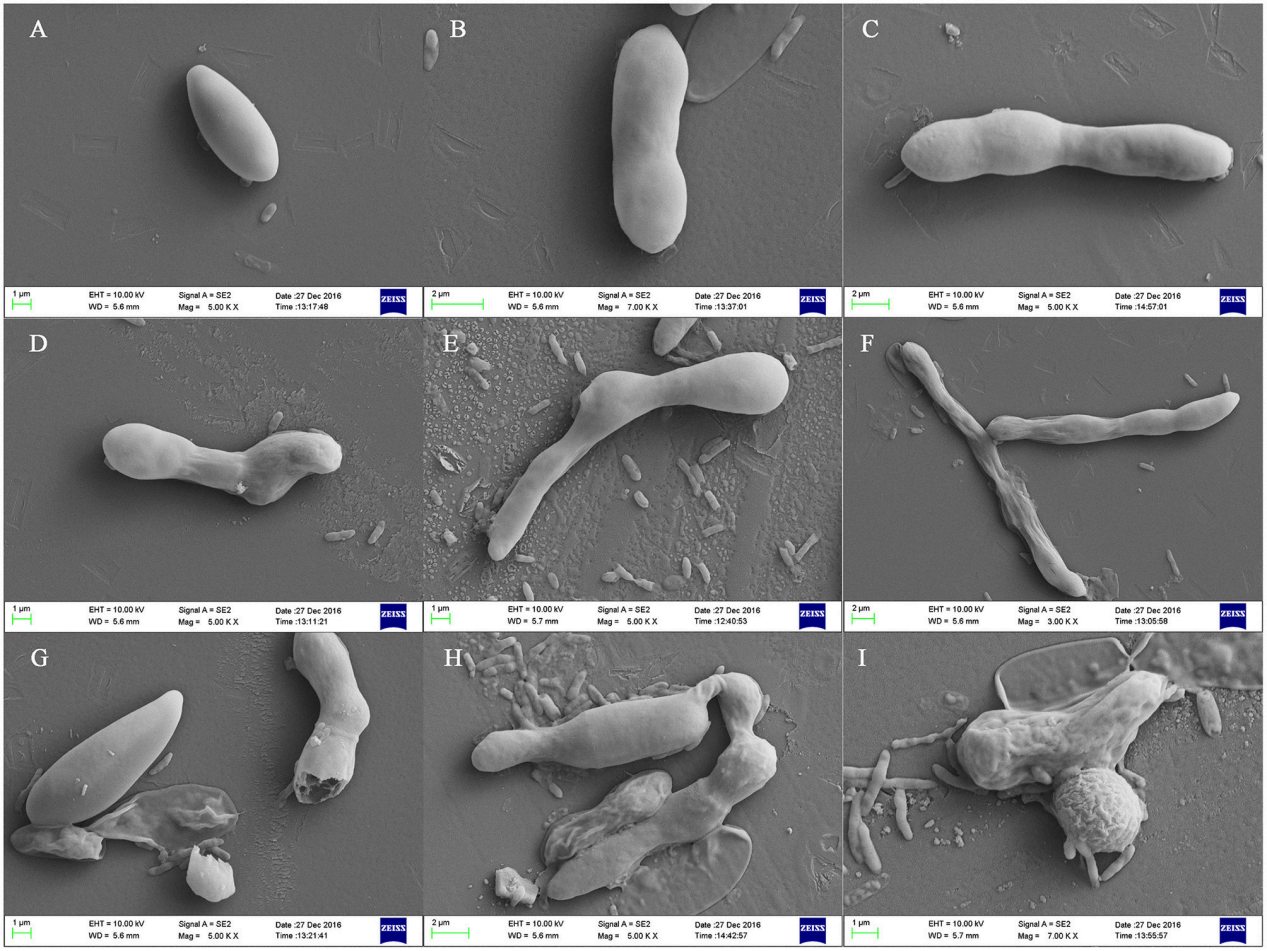
Scanning electron micrographs of *F. oxysporum* Race 4 (ATCC 76255) treated with the crude extract of *Streptomyces* sp. CB-75. **(A–C)** Treatment with 10% DMSO, **(D–I)** treatment with the crude extract of *Streptomyces* sp. CB-75.

The effect of the crude extract of *Streptomyces* sp. CB-75 against *F. oxysporum* Race 4 (ATCC 76255) was confirmed by SEM (Figure 4). SEM images of the cells treated with 2 × MIC of the crude extract revealed considerable morphological alterations, including deformation, shrinkage, collapse, tortuosity, and broken cells, leading to prominent cell shape loss and integrity (Figures 4D–I). The control cells treated with 10% DMSO were intact with smooth surfaces (Figures 4A–C).

#### GC-MS analysis

**Figure 5:**
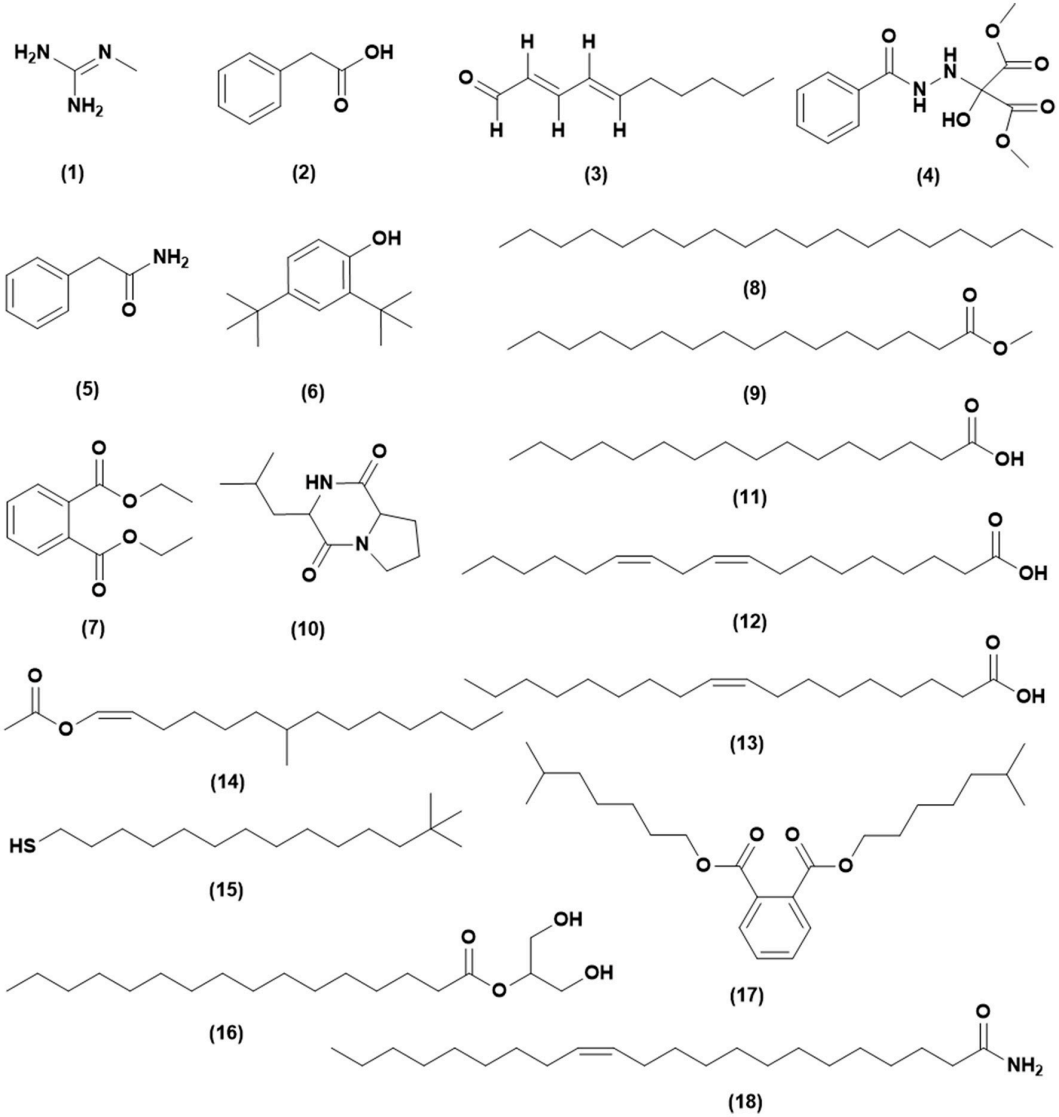
Chemical structures of the identified compounds from the crude extract of *Streptomyces* sp. CB-75.

From the GC-MS analysis, 18 chemical compounds of the crude extract of *Streptomyces* sp. CB-75 were identified by the National Institute of Standards and Technology (NIST) library based on their retention time, molecular mass, molecular formula (Table 6), and their chemical structures, listed in Figure 5. These compounds were methyl-guanidine (1), 2-phenylacetic acid (2), (*E,E*)-2,4-decadienal (3), dimethyl 2-(2-benzoylhydrazinyl)-2-hydroxypropanedioate (4), benzeneacetamide (5), 2,4-bis(1,1-dimethylethyl)-phenol (6), diethyl phthalate (7), nonadecane (8), hexadecanoic acid, methyl ester (9), 3-isobutylhexahydropyrrolo[1,2-a]pyrazine-1,4-dione (10), *n*-hexadecanoic acid (11), (*Z,Z*)-9,12-octadecadienoic acid (12), oleic acid (13), 7-methyl-*Z*-tetradecen-1-ol acetate (14), 13,13-dimethyltetradecane-1-thiol (15), hexadecanoic acid, 2-hydroxy-1-(hydroxymethyl) ethyl ester (16), 1,2-benzenedicarboxylic acid diisooctyl ester (17), and (*Z*)-13-docosenamide (18). The peak area of the compounds was in direct proportion to their quantity in the crude extract of *Streptomyces* sp. CB-75 (Figure 5).

**Table 6:** Compounds identified from the crude extract of *Streptomyces* sp. CB-75 through GC-MS.

**Compound name**	**Probability (%)**	**RT (min)**	**MM**	**Area (%)**	**MF**	**Activity**	**References**
Methyl-guanidine	58.00	7.52	73	0.65	C_2_H_7_N_3_	Cytotoxic	Bosco et al., 2017
2-Phenylacetic acid	57.31	16.64	136	6.67	C_8_H_8_O_2_	Antimicrobial	Zhu et al., 2011
(*E,E*)-2,4-Decadienal	33.67	18.46	152	0.35	C_10_H_16_O	Nematicidal	Ntalli et al., 2016
Dimethyl 2-(2-benzoylhydrazinyl)-2-hydroxypropanedioate	88.01	18.94	282	0.47	C_12_H_14_N_2_O_6_	No activity reported	
Benzeneacetamide	75.89	20.09	135	5.08	C_8_H_9_NO	Antidepressant Anticonvulsant	Guan et al., 2016
2,4-Bis(1,1-dimethylethyl)-phenol	33.06	21.74	206	0.49	C_14_H_22_O	No activity reported	
Diethyl phthalate	68.10	22.98	222	0.51	C_12_H_14_O_4_	No activity reported	
Nonadecane	21.70	24.12	268	0.62	C_19_H_40_	No activity reported	
Hexadecanoic acid, methyl ester	33.31	26.65	270	1.21	C_17_H_34_O_2_	Antioxidant	Ajoku et al., 2015
3-Isobutylhexahydropyrrolo[1,2-a]pyrazine-1,4-dione	36.39	26.91	210	1.71	C_11_H_18_N_2_O_2_	Antioxidant	Ser et al., 2015a
*n*-Hexadecanoic acid	60.58	27.03	256	5.21	C_16_H_32_O_2_	Antibacterial	Johannes et al., 2016
(*Z,Z*)-9,12-Octadecadienoic acid	16.08	28.75	280	5.78	C_18_H_32_O_2_	No activity reported	
Oleic acid	30.93	28.95	282	10.02	C_18_H_34_O_2_	No activity reported	
7-Methyl-*Z*-tetradecen-1-ol acetate	11.09	29.12	268	1.64	C_17_H_32_O_2_	No activity reported	
13,13-Dimethyltetradecane-1-thiol	17.38	29.20	258	4.04	C_16_H_34_S	No activity reported	
Hexadecanoic acid, 2-hydroxy-1-(hydroxymethyl) ethyl ester	60.79	33.55	330	0.88	C_19_H_38_O_4_	Antioxidant, anti-inflammatory, anthelmintic	Al-Marzoqi et al., 2015
1,2-Benzenedicarboxylic acid diisooctyl ester	58.19	33.85	390	0.75	C_24_H_38_O_4_	Antifungal	Rahman and Anwar, 2006
(*Z*)-13-Docosenamide	50.95	40.64	337	53.26	C_22_H_43_NO	Antiviral	Donio et al., 2013

*RT, Retention time; MM, Molecular mass of compound; MF, Molecular formula*.

#### Control effects of *Streptomyces* sp. CB-75 fermentation broth on banana *Fusarium* wilt

*Fusarium* wilt disease spread rapidly when plantlets were inoculated with a high concentration of *F. oxysporum* Race 4. However, there was no significant difference between the treatments and the control (Table 7). After 15 days, the infected plantlets were treated with the fermentation broth of CB-75. As time went on, plantlets began to die, and on the 60th day, fifteen plantlets were dead in the CK2 group, four plantlets were dead in the CK3 group, nine plantlets were dead in the A group, while there were no dead plantlets in the CK1 and AB groups. The disease indices of CK2, CK3, and A were very high (CK2 was 81.38, CK3 was 53.81, A was 70.45). The prevention and control effect of CK3 was better (51.88%). However, when the plantlets were treated with the CB-75 fermentation broth (AB), the disease index was 10.23, and the prevention and control effect was the best (83.12%).

**Table 7:** Effects of *Streptomyces* sp. CB-75 on banana *Fusarium* wilt and the growth of banana plantlets.

**Treatment**	**Disease index**	**Control effect (%)**	**Leaf area (cm^2^/plant)**	**Root length (cm)**	**Root diameter (mm)**	**Plant height (cm)**	**Stem (cm)**
CK1	–	100	1, 031.55 ± 18.82 c	1, 000.12 ± 19.74 c	0.66 ± 2.13 c	50.98 ± 4.07 b	5.71 ± 0.88 b
CK2	81.38 ± 3.17 a	–	263.96 ± 20.33 e	383.24 ± 21.51 e	0.24 ± 1.85 e	22.07 ± 4.07 e	3.64 ± 0.88 e
CK3	53.81 ± 1.85 c	51.88	1, 155.42 ± 20.08 b	1, 036.92 ± 18.66 b	0.78 ± 1.98 b	46.22 ± 3.86 c	4.97 ± 0.97 c
A	70.45 ± 2.33 b	10.45	884.77 ± 23.14 d	711.25 ± 20.45 d	0.47 ± 2.01 d	33.23 ± 5.32 d	4.10 ± 0.58 c
AB	10.23 ± 2.58 d	83.12	1, 398.13 ± 22.12 a	1, 354.87 ± 21.45 a	1.11 ± 2.32 a	53.76 ± 5.91 a	6.19 ± 0.60 a

*Data in the table are means ± SD. Different lowercase letters in the same column show values that are significantly different at the P < 0.05 level by Duncan's new multiple range test*.

#### Effect of *Streptomyces* sp. CB-75 on banana chlorophyll content

**Figure 6:**
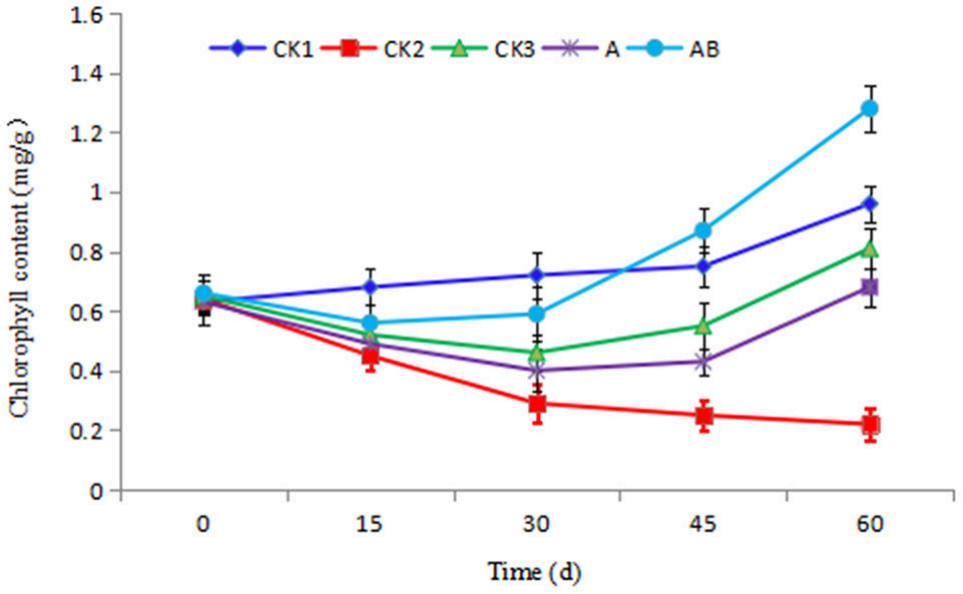
Effect of *Streptomyces* sp. CB-75 on banana chlorophyll content.

As shown in Figure 6, with increasing transplanting period, the chlorophyll content of CK1 continued to rise, the chlorophyll content of CK2 showed a decreasing trend, but the chlorophyll content of the plants in the CK3, A, and AB treatment groups first decreased and then increased. The final value of AB treatment was the highest on the 60th day, and the rising trend was also significantly higher than that of the other treatments. The chlorophyll content was negatively correlated with the incidence index. The control effect in the CK1 group was lower, with 15 dead plantlets, and the chlorophyll content also decreased with the transplanting period increased. The chlorophyll content of CK3 and A showed an increasing trend, 0.81 mg/g and 0.68 mg/g, respectively, but the final value was always lower than that of CK1 treatment. In the AB treatment group, due to the presence of bioactive compounds, the disease index of banana seedlings decreased in the latter stages, and the effect of disease prevention was significant. The chlorophyll content was 1.28 mg/g on the 60th day, which was significantly higher than for the other treatments. The chlorophyll content was 88.24% compared with A treatment, and 33.33% compared with CK1 treatment. This was indicative that the fermentation broth of CB-75 improved the chlorophyll content of banana plants.

#### Effects of *Streptomyces* sp. CB-75 fermentation broth on the growth of banana plants

After 15 days, the banana seedlings were not significantly different for leaf area, root length, root diameter, plant height, and stem among the five treatments. After 45 days, banana seedlings were treated by the fermentation broth of CB-75, the leaf area was 1,398.13 cm^2^, root length was 1354.87 cm, root diameter was 1.11 mm, plant height was 53.76 cm, and the stem was 6.19 cm, which were significantly higher than for the other treatments (Table 7). The leaf area increased by 88.24%, root length increased by 90.49%, root diameter increased by 136.17%, plant height increased by 61.78%, and stem increased by 50.98% compared with treatment A. Thus, the fermentation broth of CB-75 not only had antifungal activity, but also had a growth-promoting effect on the banana seedlings. The fermentation broth promoted plant leaf area, enhanced photosynthesis, increased root growth, and promoted transpiration, thereby promoting banana plant growth and increasing yield. Wang et al. (2016) found that the *Bacillus amyloliquefaciens* strain W19 can promote the growth and fruit yield of banana, while suppressing the banana *Fusarium* wilt disease. *Streptomycetes* sp. K2 was isolated from strawberry field soils, and promoted the growth of strawberry plants and fruits (Eccleston et al., 2010).

#### Effect of *Streptomyces* sp. CB-75 on banana biomass

**Figure 7:**
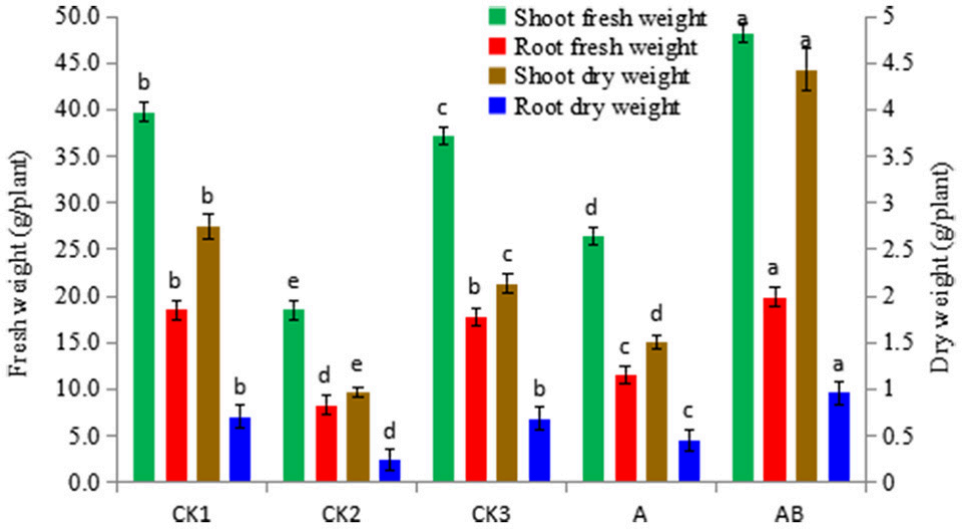
Effect of *Streptomyces* sp. CB-75 on banana fresh weight and dry weight. Different lowercase letters (a, b, c, d, e) in the figure indicate a significant difference at the *P* < 0.05 level by Duncan's new multiple range test.

The mean fresh weight and dry weight of plantlets was higher for those treated with *Streptomyces* sp. CB-75 compared with those in the other groups (Figure 7). Shoot weights (fresh and dry) was in the following order: AB>CK1>CK3>A>CK2. The weight for the AB treatment group was significantly higher than for the other treatments, shoot fresh weight was 48.20 g, shoot dry weight was 4.34 g, and water content was 90.99%. There was no significant difference in root fresh weight between AB and CK1 treatments, which were significantly higher than for the others treatment. The root dry weight for the AB treatment group was significantly higher than for the other treatments; there was no significant difference in root dry weight between CK1 and CK3. The results showed that the dry matter accumulation increased significantly, and was higher than normal for the growth of banana seedlings treated with *Streptomyces* sp. CB-75. The shoot fresh weight increased by 82.38%, the root fresh weight increased by 72.01%, the shoot dry weight increased by 195.33%, and the root dry weight increased by 113.33% compared with the treatment A group. Thus, the fermentation broth of *Streptomyces* sp. CB-75 had a growth-promoting effect on banana plants.

## Discussion

Plant diseases caused by pathogenic fungi bring about heavy losses in agriculture and result in a serious threat to global food security (Savary et al., 2006). Most losses of fruits and vegetables have been attributed to fungal pathogens, they often cause crops to rot, and sometimes produce mycotoxins that are harmful to animal and human health (Saremi and Okhovvat, 2006). *F. oxysporum* is a major pathogenic fungus that can cause postharvest decay in crops (Saremi and Okhovvat, 2006). Banana *Fusarium* wilt (caused by *F. oxysporum*) is a common plant disease that causes leaf blight; it is a devastating disease that causes significant economic damage to banana. However, the utilization of antagonistic microorganisms within the soil can effectively control the growth and reproduction of the pathogen (Deng et al., 2013). The biological control of plant diseases has become a focus of research due to its high efficiency, broad spectrum, and environmental friendliness, and because it will not cause plant pathogen resistance. However, the correct isolation and application of biocontrol microorganisms is a key factor in biological control (Chaves et al., 2011). Research shows that biofertilizer can effectively prevent plant diseases, which may be due to the presence of antagonistic microbes that become the dominant population in the micro-environment, so as to give full play to the antibacterial or bactericidal effect (Swain and Ray, 2009). Therefore, isolation and screening of highly efficient antagonistic microorganisms is the key to the development of biocontrol reagents.

Actinomycetales, especially *Streptomyces* strains, have a unique and proven capacity to produce novel antibiotics, and these species have great practical value (Hong et al., 2009). The actinomycetales *Streptomyces* sp. CB-75 was isolated using the spread plate method from the soil of a diseased banana plantation. By using 16s rRNA sequence analysis, combined with morphological, culture, physiological, and biochemical characteristics, the results showed that strain *Streptomyces* sp. CB-75 exhibited the highest similarity to the strain *Streptomyces spectabilis* NBRC 13424 (Spasova et al., 1997).

Actinomycetes produce a wide range of bioactive secondary metabolites that are known to have anti-inflammatory, antimalarial, antifungal, antibacterial, antialgal, and anticancer activities. Approximately two-thirds of available antibiotics have been isolated from actinomycetes (Wang J. J. et al., 2015). The antibiotic oligomycin A was isolated from *Streptomyces diastatochromogenes*, and was found to be active against several phytopathogenic fungi, such as *Botrytis cinerea, Cladosporium cucumerinum, Colletotrichum lagenarium, Phytophthora capsici, Alternaria alternata*, and *Aspergillus niger* (Smith et al., 1954; Kim et al., 1999; Yang et al., 2010). An antagonistic compound was isolated from *Streptomyces* sp. TP-A0595 and identified as 6-prenylindole, with suppressive effect on infection by *Alternaria brassicicola* by inhibiting the formation of infection hyphae (Sasaki et al., 2002). Two compounds were purified from *Streptomyces* sp. 3-10, and were identified as reveromycins A and B, which demonstrated high antifungal activity against *Botrytis cinerea, Mucor hiemalis, Rhizopus stolonifer*, and *Sclerotinia sclerotiorum* (Lyu et al., 2017). Srivastava et al. found *Chrestomyceticus* could produce metabolites with antifungal activity against *Candida albicans* (Srivastava and Dubey, 2016). Nguyen et al. isolated a monomer compound from *Streptomyces griseus* H7602 with strong inhibitory activity against *Phytophthora capsici*, and the antibacterial mechanism was studied (Nguyen et al., 2015). Some well-known antibiotics produced by *Streptomyces* have been used as fungicides. For instance, blasticidin-S was isolated from *Streptomyces griseochromogenes* and was an antibiotic commercially introduced for the control of rice blast in Japan (Fukunaga et al., 1955; Tapadar and Jha, 2013). *Streptomyces spectabilis* can produce many types of antibiotics with high antibacterial activity, including spectinomycin (Kim et al., 2008), streptovaricin (Kakinuma et al., 1976), and desertomycin (Ivanova, 1997), and has a high application value in the pharmaceutical industry (Selvakumar et al., 2015). However, there are no reports on its activity against soil-borne plant diseases and it isn't currently used in agriculture. The discovery of this *Streptomyces* strain provides new products for the pharmaceutical industry, but also provides a method for the control of plant diseases, and lays the foundation for future agricultural studies. In the future, the extraction and purification of the antifungal metabolites will be studied, and the antifungal mechanism will be improved and perfected.

Biosynthetic gene clusters are responsible for microbial natural product biosynthesis. Polyketides and non-ribosomal peptides are a structurally varied group of compounds, and play important biological roles (Nimaichand et al., 2015). The genes encoding PKS-I and NRPS might both play a part in the production of antifungal activity from *Streptomyces* sp. CB-75. The results were similar to the studies of Sharma et al. (2016) and Passari et al. (2015), and it was shown that actinomycetes possessing antifungal activity were positive for the presence of both of these two biosynthetic pathway genes in their genomes. The presence of genes encoding PKS-I and NRPS in strain CB-75 is indicative of the possibility that it can produce bioactive secondary metabolites belonging to these two classes of natural products, or a hybrid of both (Dhaneesha et al., 2017). Relatively low sequence similarity of the PKS-I gene sequences (84%) with those available in GenBank is indicative of the possibility that novel compounds are produced by *Streptomyces* sp. CB-75.

GC-MS is a powerful analytical tool for the chemical analysis of microbial metabolites (Ser et al., 2015a,b; Tan et al., 2015). GC-MS plays a significant role in natural product discovery, including bioactive compounds derived from *Streptomyces* species (Ara et al., 2014; Jog et al., 2014). Ser et al. (2015a) reported the detection of an antioxidative bioactive compound, 3-Isobutylhexahydropyrrolo[1,2-a]pyrazine-1,4-dione, in an extract of *Streptomyces mangrovisoli* sp. nov., and Kim et al. (2008) found protocatechualdehyde in an *Streptomyces lincolnensis* M-20 extract using GC-MS. GC-MS analysis was performed on a crude extract of *Streptomyces* sp. CB-75, and 18 chemical compounds were found with different retention times and relative abundances. The compounds present included an alkaloid, polymeric aldehyde, hydrocarbons, acids, terpenoids, phenolic, esters, and quinones. Methyl-guanidine is a known nephrotoxin and neurotoxin, and increases oxidative metabolism and accelerates apoptosis of neutrophils (Noda et al., 2016; Bosco et al., 2017). 2-Phenylacetic acid was found to have effective antibacterial activity against *Escherichia coli* and *Ralstonia solanacearum* (Zhu et al., 2011). (*E,E*)-2,4-Decadienal exhibited strong paralytic activity on second-stage *Meloidogyne incognita, Meloidogyne javanica* and *Meloidogyne arenaria* juveniles (Ntalli et al., 2016). Guan et al. (2016) reported benzeneacetamide as an antidepressant-like and anticonvulsant compound. Phenolic compounds were alluded to as potent antimicrobial agents, as are free radical terminators, as they possess hydrogen-donating capability to reduce free radicals (Yogeswari et al., 2012). Recently, a study conducted by Kumar P. S. et al. (2014) showed high antimicrobial activity in the GC-MS fractions containing the highest amounts of phenolic compounds. Phenolic compounds, such as 2,4-bis(1,1-dimethylethyl)-phenol, were detected in CB-75. Hexadecanoic acid methyl ester has been reported to cause autolysis of membranous structures, induce significant aortic dilation, inhibit phagocytic activity and nitric oxide production of certain cells, reduce levels of tumor necrosis, and was isolated from the *Hibiscus sabdariffa* Linn (Ajoku et al., 2015). According to recent reports by Ser et al. (2015a), 3-Isobutylhexahydropyrrolo[1,2-a]pyrazine-1,4-dione exhibited strong antioxidant activity and was useful as a preventive agent against free-radical-associated diseases. Hexadecanoic acid was shown to have antibacterial activity by damaging the cell walls of *Salmonella typhi* (Johannes et al., 2016). Al-Marzoqi et al. (2015) reported antioxidant, anti-inflammatory and anthelmintic activities of hexadecanoic acid, 2-hydroxy-1-(hydroxymethyl) ethyl ester. 1,2-Benzenedicarboxylic acid diisooctyl ester, isolated from the roots of *Plumbago zeylanica* was screened for its antifungal activity against six phytopathogenic fungi (Rahman and Anwar, 2006). Another study conducted by Donio et al. (2013) illustrated that (*Z*)-13-docosenamide isolated from halophilic *Bacillus* sp. BS3 possesses strong antiviral activity. These compounds are well recognized for their antifungal activity and together they may be responsible for the broad-spectrum antifungal activity of CB-75 against the wide range of test fungal pathogens. Previous reports by Sharma et al. (2016), Tan et al. (2015), and Ser et al. (2015a,b) showed the common effect of bioactive compounds from GC-MS analysis. Thus, we propose that these compounds could be the key contributing factors to the antifungal activities of CB-75. The study of other biological activities of the metabolites produced by strain CB-75 is the subject of further investigation.

In recent years, the research of actinomycetes was focused on its ability to control plant disease and indirectly promote plant growth. Most isolates in the genus *Streptomyces* showed surpassing antifungal activities against fungal pathogens, and abilities to produce plant-growth-promoting agents in high quantity (Himaman et al., 2016). Actinomycetes could provide nutrients by the specific uptake system to stimulate plant growth (Rungin et al., 2012). Mahadevan and Crawford (1997) found *Streptomyces olivaceoviridis, Streptomyces rimosus, Streptomyces rochei, Streptomyces griseoviridis*, and *Streptomyces lydicus* had the ability to improve plant growth by increasing seed germination, root elongation, and root dry weight. Uphoff et al. (2009) reported *Streptomyces* strains significantly enhanced plant growth by increasing plant root length, number of roots, plant shoot length, number of leaves, fresh weight, and dry weight over the un-inoculated control. Almost all the rhizospheric actinomycetes were also able to produce ammonia and hydrogen cyanide (Anwar et al., 2016). Marques et al. (2010) found that bacteria could synthesize ammonia and supply nitrogen to the host plant. Furthermore, overproduction of ammonia serves as an inhibition factor for the plant pathogens. Ammonia and hydrogen cyanide production also play an important role in suppression of plant disease. Hastuti et al. (2012) reported *Streptomyces* sp. LSW05 can produce hydrogen cyanide. Anwar et al. (2016) found that *Streptomyces kunmingensis* WC-3, *Streptomyces enissocaesilis* TA-3, *Streptomyces* sp. WA-1, and *Streptomyces djakartensis* TB-4 could produce ammonia and hydrogen cyanide. These rhizospheric *Streptomyces* were good candidates to be developed as biofertilizers for growth promotion and yield enhancement in crops, and could be exploited for the commercial production of different agro-active compounds. The study had demonstrated for the first time, to our knowledge, that the selected *Streptomyces* sp. CB-75 had strong antagonistic ability against *F. oxysporum* by pot experiments. This strain had a good control effect on banana *Fusarium* wilt (83.12%). It could increase banana leaf area, root length, root diameter, plant height, stem, and biomass. The findings from this current study are clearly indicative of the possibilities of using *Streptomyces* sp. as a bio-inoculant for growth promotion, nutrient mobilization, and biocontrol in banana seedling production.

## Conclusion

During the exploration of antagonistic actinomycete predominant in soil samples of a diseased banana plantation in Hainan, China, *Streptomyces* sp. CB-75 was isolated by serial dilution technique. Based on phenotypic and molecular characteristics, and 99.93% sequence similarity with *Streptomyces spectabilis* NBRC 13424 (AB184393), the strain was identified as *Streptomyces* sp. This strain exhibited broad-spectrum antifungal activity against 11 plant pathogenic fungi. Type I polyketide synthase (PKS-I) and non-ribosomal peptide synthetase (NRPS) were detected, which were indicative of the antifungal compounds that *Streptomyces* sp. CB-75 could produce. An ethyl acetate extract from the strain exhibited the lowest minimum inhibitory concentration (MIC) against *Colletotrichum musae* (ATCC 96167) (0.78 μg/ml) and yielded the highest antifungal activity against *Colletotrichum gloeosporioides* (ATCC 16330) (50.0 μg/ml). Also, spore germination was significantly inhibited by the crude extract. After treatment with the crude extract of *Streptomyces* sp. CB-75 at the concentration 2 × MIC, the pathogenic fungi showed deformation, shrinkage, collapse, and tortuosity when observed by scanning electron microscopy (SEM). By gas chromatography-mass spectrometry (GC-MS) of the crude extract, 18 chemical constituents were identified; (*Z*)-13-docosenamide was the major constituent. Pot experiments showed that the incidence of banana seedlings was reduced after using *Streptomyces* sp. CB-75 treatment. The disease index was 10.23, and the prevention and control effect was 83.12%. Furthermore, *Streptomyces* sp. CB-75 had a growth-promoting effect on banana plants. The chlorophyll content showed 88.24% improvement, the leaf area, root length, root diameter, plant height, and stem showed 88.24, 90.49, 136.17, 61.78, and 50.98% improvement, respectively, and the shoot fresh weight, root fresh weight, shoot dry weight, and root dry weight showed 82.38, 72.01, 195.33, and 113.33% improvement, respectively, compared with treatment of fermentation broth without *Streptomyces* sp. CB-75. Thus, *Streptomyces* sp. CB-75 is an important microbial resource as a biological control against plant pathogenic fungi and for promoting banana growth. From the results, it is obvious that *Streptomyces* sp. CB-75 is a promising candidate for the development of potential antifungal biocontrol agents against a wide range of fungal pathogens, and could be exploited as a fungicide to control plant fungal diseases. Moreover, more work is needed to optimize formulation, fermentation conditions, and application methods of *Streptomyces* sp. CB-75, in order to fully maximize its potential as an effective agent for controlling plant diseases.

## Author contributions

YC and YL designed the research. YL and JX supervised the research work and guided the experimental design. DZ provided the suggestion of the research work. YC, DZ, ZG, and DQ were involved in soil sampling, DNA extraction, and amplification. YC and DZ conducted the other experiments. YC analyzed the data and was involved with writing the paper. YC and YL prepared the manuscript.

### Conflict of interest statement

The authors declare that the research was conducted in the absence of any commercial or financial relationships that could be construed as a potential conflict of interest.

## Abbreviations

DMSO: Dimethyl sulfoxide
EtOAc: ethyl acetate
GC-MS: gas chromatography-mass spectrometry
MIC: minimum inhibitory concentration
NRPS: non-ribosomal peptide synthetase
PKS: polyketide synthase
SEM: scanning electron microscopy.
